# The Diagnostic Value of Alpha-1-Antitrypsin Phenotype in Patients with Granulomatosis with Polyangiitis

**DOI:** 10.1155/2016/7831410

**Published:** 2016-04-10

**Authors:** M. Y. Pervakova, V. L. Emanuel, O. N. Titova, S. V. Lapin, V. I. Mazurov, I. B. Belyaeva, A. L. Chudinov, T. V. Blinova, E. A. Surkova

**Affiliations:** ^1^First Pavlov State Medical University of St. Petersburg, Saint Petersburg 197022, Russia; ^2^North-Western Mechnikov State Medical University, Saint Petersburg 191015, Russia

## Abstract

The deficiency of alpha-1 protease inhibitor, or alpha-1-antitrypsin (A1AT), predisposes to chronic lung diseases and extrapulmonary pathology. Besides classical manifestations, such as pulmonary emphysema and liver disease, alpha-1-antitrypsin deficiency (A1ATD) is also known to be associated with granulomatosis with polyangiitis (GPA or Wegener's granulomatosis). The aim of our study was to evaluate the frequency of allelic isoforms of A1AT and their clinical significance among GPA patients. Detailed clinical information, including Birmingham Vasculitis Activity Score (BVAS), incidence of lung involvement, anti-proteinase 3 (PR3) antibodies concentrations, and other laboratory data were collected in 38 GPA patients. We also studied serum samples obtained from 46 healthy donors. In all collected samples A1AT phenotyping by isoelectrofocusing (IEF) and turbidimetric A1AT measurement were performed. Abnormal A1AT variants were found in 18.4% (7/38) of cases: 1 ZZ, 4 MZ, 2 MF, and only 1 MZ in control group (2%). The mean A1AT concentration in samples with atypical A1AT phenotypes was significantly lower (*P* = 0.0038) than in normal A1AT phenotype. We found that patients with abnormal A1AT phenotypes had significantly higher vasculitis activity (BVAS) as well as anti-PR3 antibodies concentration. We conclude that A1AT deficiency should be considered in all patients with GPA.

## 1. Introduction

Granulomatosis with polyangiitis (Wegener's, GPA) is a disease manifested by necrotizing granulomatous inflammation affecting predominantly small to medium vessels and associated with presence of antineutrophil cytoplasmic antibodies (ANCA) in blood. Upper respiratory tract, eyes, lungs, and kidneys are typical target organs; rarely skin, joints, and nervous system are also involved [1]. This type of vasculitis is rare (annual frequency in Northern Europe is lower than 1 : 100000), but quite aggressive disease, which results in lethal outcome in 90% of cases during first year if left untreated [2]. An elevated frequency of alpha-1-antitrypsin (A1AT) phenotypic variants was found in GPA patients in comparison with population incidence [3].

A1AT is an acute phase protein belonging to the serine proteases inhibitors family and is capable of inactivating many proteases including proteinase 3 that is recognized as the main autoantigenic target in GPA (PR3) [4]. Alpha-1-antitrypsin deficiency (A1ATD) is a frequent genetic disorder caused by low serum A1AT concentration as a result of carriage of pathogenic alleles of Pi-gene (protease inhibitor) [5]. The deficiency of this important protective factor leads to different types of lung tissue injury, such as emphysema or destructive inflammation in GPA [6].

The most common and normally functioning A1AT allelic form is PiM, so healthy human phenotype is designated as PiMM. There are more than 100 genetic A1AT variants, among which PiZ and PiS are the most common and clinically significant. A1ATD becomes clinically manifested in individuals carrying mutation in both gene Pi alleles, especially in PiZZ variant, whereas in heterozygous state the defect is partly compensated by normal allele that is found in individuals with PiMZ and PiMS phenotypes [7]. Heterozygous A1AT carriage does not provide high risk of A1ATD, though it predisposes to some diseases, including GPA [8].

According to the statistical data of American Thoracic Society/European Respiratory Society (ATS/ERS), the frequency of Z-alleles in GPA patients in Europe varies from 9 to 17.6% [9]. Quantitative methods, like turbidimetric measurement, are generally used for the laboratory detection of A1AT [10]. However, the diagnostic efficacy of these assays is limited, because the result might be incorrect due to cross-reactivity with lipids or haemoglobin [9], or acute phase reaction [11]. Reference method of screening for A1ATD is isoelectrofocusing (IEF) [12, 13] with selective A1AT staining with polyclonal anti-A1AT antibodies [14, 15].

*The aim* of our study was to evaluate the frequency of pathogenic A1AT alleles among Russian GPA patients and to find out whether A1AT phenotype influences vasculitis activity.

## 2. Materials and Methods

Serum samples and clinical data were provided by Saint-Petersburg Clinical Rheumatology Hospital number 25. 38 sera were obtained from individuals, suffering from active anti-PR3-positive GPA. 46 samples of healthy blood donors were collected as a control group. To estimate the clinical significance of different A1AT phenotypic variants, we collected detailed clinical and laboratory data, including Birmingham Vasculitis Activity Score (BVAS), the incidence of lung involvement, and anti-PR3 antibodies concentrations, measured by enzyme-linked immunosorbent assay (ELISA) with a commercial kit (Euroimmun, Germany).

We determined A1AT phenotypes in all collected samples by IEF with immunoblotting with the use of horizontal electrophoresis system (Pharmacia, Sweden). PH gradient was created by adding narrow specter ampholytes pH 4.2–4.9 (GE Healthcare, Sweden). The A1AT molecules focused within agarose gel were blotted onto nitrocellulose paper and selectively stained by horseradish peroxidase conjugated goat anti-A1AT antibodies (Bethyl Laboratories, Sweden). A1AT phenotypes were assessed by comparing A1AT migration patterns with control PiMM, PiMZ, and PiMS samples.

**Figure 1:**
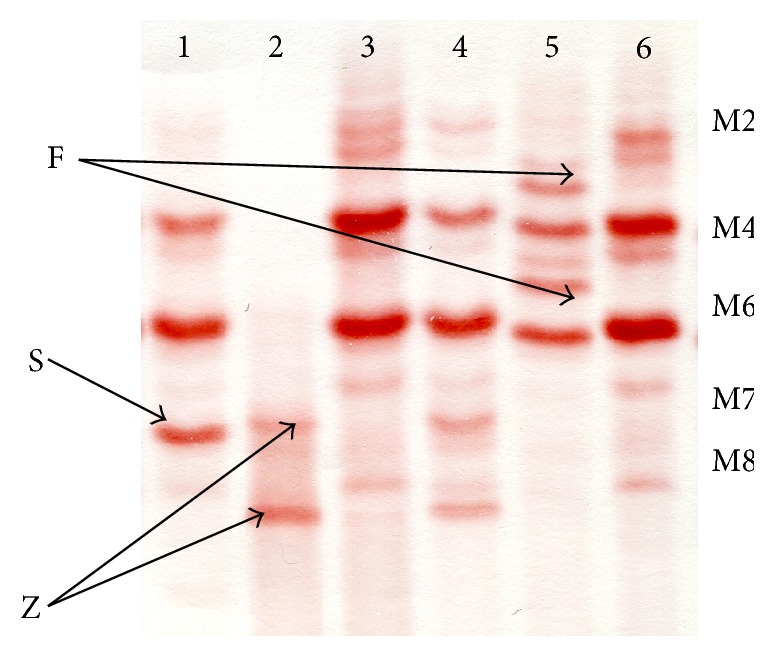
Examples of A1AT phenotype patterns, obtained by IEF. Track 1: PiMS. Track 2: PiZZ. Track 3: PiMM. Track 4: PiMZ. Track 5: PiMF. Track 6: PiMM. А1АТ: alpha-1-antitrypsin. IEF: isoelectrofocusing. M2, M4, M6, M7, and M8: zones of migration of main A1AT isoforms in normal PiMM phenotype, detected by IEF. F, Z, and S: additional bands with another migration, indicating the presence of PiF, PiS, and PiZ alleles.

Electrophoretic patterns of main A1AT isoforms migration by IEF are demonstrated in Figure 1.

Besides phenotyping, quantitative A1AT measurements were performed with turbidimetric commercial kit (Sentinel Diagnostics, Italy).

To estimate statistical significance we compared clinical and laboratory parameters of the groups, using unpaired *t*-test or Mann-Whitney* U* test, depending on whether the distribution was Gaussian. All categorical variables were compared with exact Fisher test. Differences between the groups were considered to be significant at a *P* value of <0.05. Statistical analysis was performed using Graph Pad Prism 4.0 software.

## 3. Results

We observed 38 GPA patients receiving inpatient treatment at specialized rheumatologic clinic. The group was heterogeneous by gender (16 men and 22 women) and by age (18–77 years old). All samples were anti-PR-3 ANCA-positive.

Pulmonary involvement in vasculitis was found in 64.5% (20/31) of cases and was presented by cavitating infiltrates (*N* = 13) and interstitial fibrosis (*N* = 7). All patients had a different degree of vasculitis activity, which was evaluated with BVAS clinical scale. In GPA patients, we found 18.4% (7/38) pathologic A1AT variants. Following A1AT phenotypes were identified: 4 MZ, 1 ZZ, and 2 MF. Among 46 samples of healthy donor's sera 1 heterozygous PiMZ phenotype was found.

Some clinical and laboratory parameters of 7 GPA patients, carrying pathological A1AT phenotypes, are demonstrated in Table 1.

**Figure 2:**
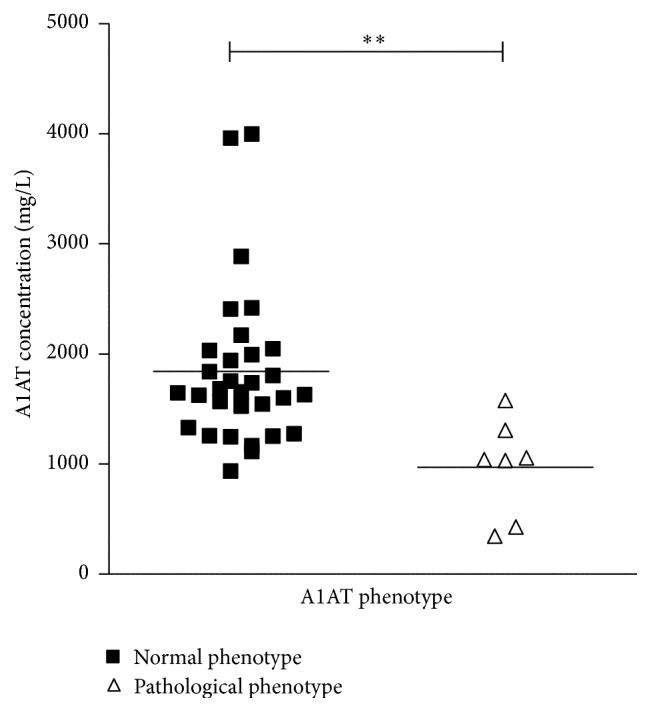
The results of quantitative A1AT measurement in serum samples of GPA patients with normal and pathological A1AT phenotypes; ^*∗∗*^ *P* < 0.01. Note: A1AT reference values: 900–2000 mg/L.

To find out clinical significance of pathogenic A1AT allelic forms, we analyzed GPA patients depending on A1AT phenotype. Mean A1AT concentrations in groups with normal (*N* = 31) and pathological (*N* = 7) A1AT phenotypes were 1840 mg/L ± 127.2 and 970.0 mg/L ± 167.6, respectively (*P* = 0.0038, unpaired *t*-test). A1AT concentrations in both groups are presented in Figure 2.

Only in 2 from 7 samples with abnormal A1AT phenotype the A1AT concentrations were below the reference range of 900 mg/L.

**Figure 3:**
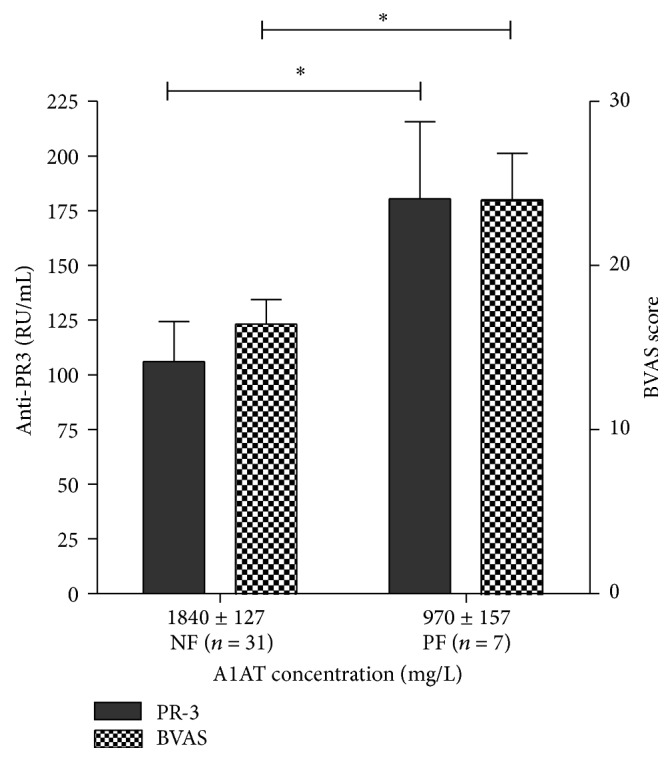
A1AT concentration, anti-PR3 antibodies concentration, and GPA activity in patients with normal and abnormal A1AT phenotypes; ^*∗*^ *P* < 0.1. NF-patients with normal A1AT phenotype; PF-patients with pathological A1AT phenotype; A1AT: alpha-1-antitrypsin; PR3: Proteinase 3, anti-PR3: anti-proteinase 3 antibodies; GPA: Granulomatosis with polyangiitis; BVAS: Birmingham Vasculitis Activity Score.

Comparing A1AT phenotypes and clinical data, we found that the mean vasculitis activity, measured with BVAS, in the group with normal phenotypes was 16.42 ± 1.498 that was significantly lower than 24.00 ± 2.828, the mean BVAS score in GPA patients with abnormal A1AT (see Figure 3).

Concentrations of anti-PR3 antibodies were also significantly higher (*P* = 0.0480, Mann-Whitney* U* test) in GPA patients with abnormal A1AT phenotypes (180.4 RU/mL ± 35.19 and 106.0 RU/mL ± 18.25, resp.).

Mean values of erythrocyte sedimentation rate (ESR) in patients with abnormal A1AT phenotypes were also statistically higher than in patients with normal phenotypes: 35.70 mm/h ± 3.278 and 55.67 mm/h ± 2.031, respectively, *P* = 0.0054. The differences in other inflammation markers, such as fibrinogen, C-reactive protein, IgG, circulating immune complexes, and complement C3 did not reach statistical significance.

We noted that pulmonary involvement was found in all GPA patients with abnormal A1AT phenotype (*N* = 7) and only in 65% (20/31) patients with normal A1AT, although this difference was not statistically significant.

## 4. Discussion

To analyze the incidence of abnormal phenotypic variants of A1AT among Russian GPA patients, we studied 38 serum samples from individuals suffering from this disease. We determined A1AT phenotype and measured its concentration in all serum samples. We found abnormal A1AT phenotypes in 18.4% of cases, over the number shown in other publications: 9–17.6% [9].

We found higher BVAS activity, greater concentrations of anti-PR3 antibodies, and higher ESR in GPA patients carrying atypical alleles of A1AT. These data suggest that GPA is more severe in these individuals and more effective treatment in such cases may be considered.

Our findings can be explained by the fact that A1AT modulates PR3 activity and therefore it is presumably an important protective factor in systemic vasculitis. Tissue inflammation induces PR3 expression by neutrophils; this is attended by oxidative burst and partial neutrophil degranulation. PR3 also stimulates IL-8 production by endotheliocytes and monocytes and promotes increase of eicosanoids level and release of leucotriene B4, launching aggressive necrotizing inflammatory cascade in GPA [16–18]. It was experimentally proved that ANCA inhibit inactivation of PR3 by A1AT molecule, when bound to PR3 in GPA patient's serum [19]. Thus PR3 exhibits proinflammatory activity; meanwhile A1AT antagonizes PR3, influencing both PR3 itself and PR3 induced neutrophil chemotaxis.

The interaction of A1AT and PR3 may have a considerable influence on severity of inflammatory process in GPA and can be impaired in case of abnormal A1AT phenotype.

The case of successful A1AT replacement treatment of individual suffering from GPA and A1ATD has been reported. When this patient with A1ATD and GPA, resistant to standard therapy, was given replacement therapy with A1AT, she developed stable remission of GPA with regression of skin lesions and improvement of pulmonary parameters [20].

## 5. Conclusions

The laboratory testing for pathological A1AT alleles is not usually ordered for patients with systemic vasculitides in routine clinical practice. Considering relatively high frequency of pathological A1AT phenotype carriage among GPA patients and its clinical significance we may conclude that the relevance of A1ATD is underestimated. During medical examination of every GPA patient, the possibility of A1AT deficiency should be considered.

## Figures and Tables

**Table 1:** The description of clinical data of GPA patients with pathological A1AT phenotypes.

Number	1	2	3	4	5	6	7

Gender	Male	Male	Male	Female	Female	Female	Female

Age	31	19	49	62	52	58	67

Disease duration (years)	8	1	3	1	1	1	8

Lung involvement type	Cavitating infiltrates	Cavitating infiltrates	Interstitial fibrosis	Cavitating infiltrates	Cavitating infiltrates	Cavitating infiltrates	Interstitial fibrosis

BVAS activity	28	26	14	20	28	38	14

A1AT phenotype	MZ	ZZ	MZ	MF	MZ	MF	MZ

A1AT concentration (mg/L)^*∗*^	1041	345	1033	1057	430	1576	1308

Anti-PR3-antibodies concentration (RU/mL)^*∗∗*^	25.0	260.5	135.1	116.0	266.0	330.9	129.4

ESR (mm/hr)	54	57	46	54	63	60	55

Note:

^*∗*^A1AT reference values: 900–2000 mg/L.

^*∗∗*^Anti-PR3 antibodies reference values: <20 RU/mL.

GPA, granulomatosis with polyangiitis; BVAS, Birmingham Vasculitis Activity Score; A1AT, alpha-1-antitrypsin; PR3, proteinase 3; ESR, erythrocyte sedimentation rate.
